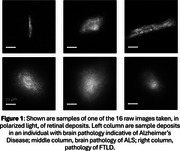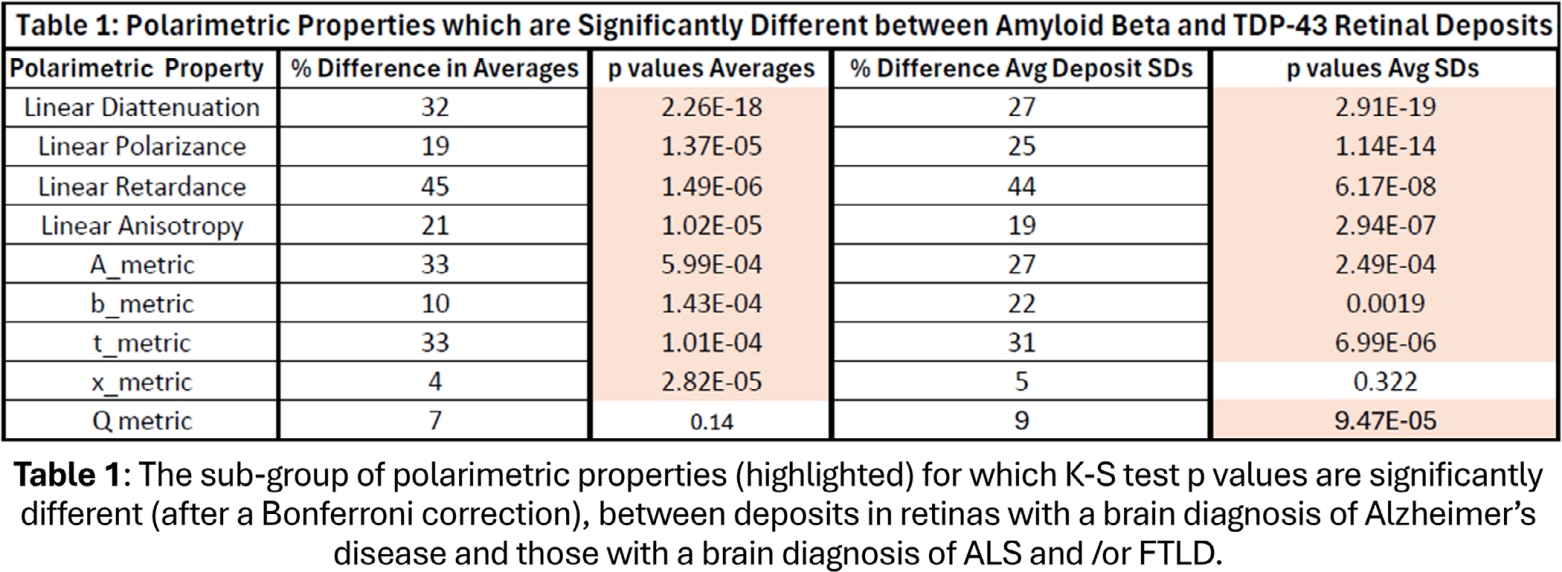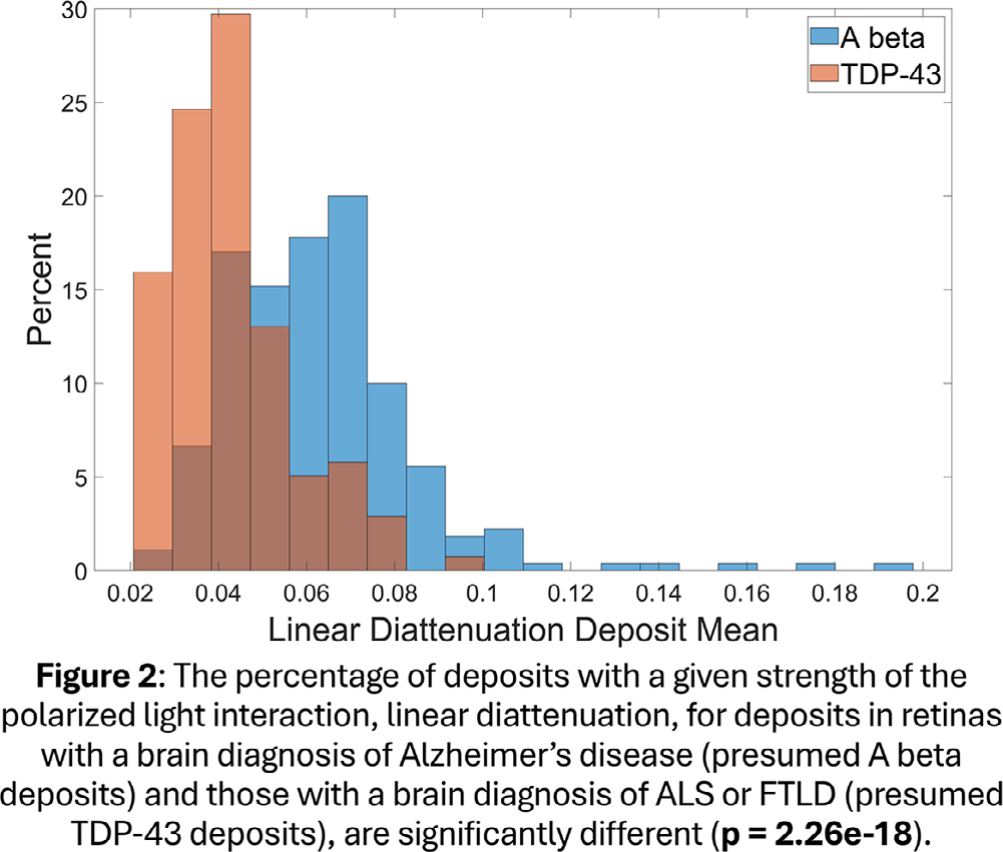# Dye Free Polarized Light Differentiation of Protein Deposits in Post‐mortem Retinas of Individuals with FTLD and ALS compared to those with Alzheimer's Disease

**DOI:** 10.1002/alz70856_099161

**Published:** 2025-12-24

**Authors:** Melanie CW Campbell, Lyndsy Acheson, Erik L Mason, Tanya Hareesha Shetty, Laura Emptage, Rachel Redekop, Monika Kitor, Ian R MacKenzie, Naomi C Futhey, Veronica Hirsch‐Reinshagen, Ging‐Yuek Robin Hsiung

**Affiliations:** ^1^ University of Waterloo, Waterloo, ON, Canada; ^2^ University of British Columbia, Vancouver, BC, Canada

## Abstract

**Background:**

Our non‐invasive retinal imaging, using polarized light, identifies protein deposits which predict brain pathology. Previously, our measured polarized light interactions differed between retinal deposits in Alzheimer's disease (AD) and neurodegenerative diseases (NDDs) involving alpha‐synuclein. Here we report deposit polarized light interactions in NDD's, involving TDP‐43, compared with amyloid deposits in AD. This inexpensive, dye‐free, differential diagnostic would be accessible to underserved populations. It would also enable appropriate, early treatments and interventions.

**Method:**

Eyes and brains were donated by 2 individuals with ALS, including 1 with concurrent FTLD, and 4 individuals with only FTLD, including 1 with Type C. TDP‐43 was present in the brains and some had additional agerelated tau. 10 individuals had amyloid beta brain pathology and a moderate to high likelihood of AD. Retinas were fixed and flat‐mounted. 270 presumed amyloid beta deposits in retinas of those with AD, and 138 presumed TDP‐43 deposits in those with FTLD and/or ALS were imaged in polarized light. For each deposit imaged, differing interactions with polarized light were calculated. In 1 case of ALS with concurrent low values of brain amyloid and 1 of FTLD‐Type C, only thioflavin negative deposits were classed as potential TDP‐43 deposits.

**Result:**

Presumed amyloid beta, consistent with AD, and TDP‐43 deposits, consistent with FTLD and/or ALS, were visible in polarized light in retinas with the corresponding brain pathologies (Figure 1). The thioflavin positive deposits from the retina with FTLD type C had polarized light interactions, not significantly different from AD deposits, consistent with protein fibrils combining TDP‐43 and the amyloid protein, ANXA11. The deposit means and/or standard deviations of nine different polarized light interactions were significantly different between the AD and TDP‐43 retinal deposits (Table 1). However, the distributions of the strengths of polarized light interactions and deposit areas overlapped between deposit types (Figure 2).

**Conclusion:**

Significant differences in many polarized light interactions are consistent with the known physical properties of the different brain (and retinal) amyloid and non‐amyloid deposits. Combinations of these interactions are expected to give early, non‐invasive, less expensive, differential diagnoses of neurodegenerative brain diseases, including those associated with amyloid beta, alpha‐synuclein and TDP‐43.